# Evaluation of Etiological Factors Associated With Abnormal Uterine Bleeding Among Teenage Girls in a Tertiary Care Center: A Cross-Sectional Study

**DOI:** 10.7759/cureus.68268

**Published:** 2024-08-31

**Authors:** Vimalambigai K, Sasikala Ramaraj, Logeswari B M, Amirtha C

**Affiliations:** 1 Obstetrics and Gynecology, Aarupadai Veedu Medical College and Hospital, Puducherry, IND; 2 Obstetrics and Gynecology, Sree Balaji Medical College and Hospital, Chennai, IND; 3 Obstetrics and Gynecology, Vinayaka Missions Medical College and Hospital, Karaikal, IND

**Keywords:** teenage girl, anovulatory dysfunction, polycystic ovary syndrome (pcos), menstrual disorders, hyperandrogenism, abnormal uterine bleeding

## Abstract

Background: Abnormal uterine bleeding (AUB) refers to irregularities in the frequency, regularity, duration, and volume of menstrual cycles, impacting about one-third of women at some point in their lives, during menarche and perimenopausal periods. This study aims to evaluate the various causes of menstrual disorders in teenage girls.

Materials and methods: This cross-sectional study was conducted in the Department of Obstetrics and Gynecology at AVMCH, Pondicherry, with ethical clearance. It included 150 girls aged 13-18 years who presented with menstrual disorders. Data were collected through structured interviews and clinical evaluations, focusing on menstrual history, socioeconomic status, associated symptoms, and investigations. Statistical analysis was performed using IBM SPSS Statistics for Windows, Version 23 (Released 2015; IBM Corp., Armonk, New York, United States).

Results: Among the etiological factors, 61.3% of patients had anovulatory dysfunction, 16.6% had early onset of PCOS, 6% of patients had hypothyroidism, 16% had ovulatory dysfunction, and 0.6% had coagulation disorder. Overall, ovulatory dysfunction (AUB-O) was predominant in teenage girls.

Conclusion: This study helps in the early identification of the etiology of adolescent AUB and the appropriate management of menstrual disorders to improve well-being and enhance reproductive function in the future.

## Introduction

Abnormal uterine bleeding (AUB) is a comprehensive term that refers to bleeding from the uterine corpus that is abnormal in frequency, regularity, duration, and volume [1]. A regular menstrual cycle typically lasts 21 to 35 days, continues for four to eight days, and results in blood loss ranging from 30 to 80 milliliters. Deviations in any of these four characteristics are considered abnormal menstrual bleeding (AUB). One-third of all women will experience irregularities at some point in their lives, including menarche, adolescence, reproductive age groups, perimenopausal, or even post-menopausal (bleeding in the post-menopausal period is considered abnormal).

AUB is defined as irregular bleeding from the uterine corpus in terms of duration, volume, frequency, and regularity [2]. Anovulation is frequently observed for approximately two years after menarche due to the immature hypothalamo-pituitary-ovarian axis. Additional causes of AUB in teenage girls include bleeding disorders, coagulation and platelet abnormalities, thyroid dysfunction, early-onset polycystic ovary syndrome (PCOS), atypical congenital adrenal hyperplasia, and, in rare cases, estrogen-secreting tumors such as granulosa cell tumors [3-6].

Young girls are often hesitant to discuss menstrual bleeding issues. When they do, the condition can be sensitive and distressing for both the adolescents and their parents, who may not understand what constitutes a normal menstrual pattern. Teenage girls may experience heightened anxiety, depression, and decreased self-esteem due to the unpredictable nature of their menstrual cycles. The stigma surrounding menstruation in many cultures further exacerbates these issues, making it difficult for young girls to seek help and receive appropriate care [2]. The primary goal is to identify any underlying medical conditions that may have long-term health implications and to alleviate the anxiety of both the affected girls and their families.

In 2011, the International Federation of Gynecology and Obstetrics proposed a new classification for AUB known as PALM-COEIN. This classification identifies structural causes as PALM (Polyp, Adenomyosis, Leiomyoma, and Malignancy) [7], and non-structural causes as COEIN (coagulopathy, ovulatory dysfunction, endometrial, iatrogenic, and not otherwise classified). In adolescents, AUB is mainly due to non-structural causes. Additionally, ethical and cultural considerations must be taken into account, as different cultural backgrounds influence how menstrual issues are perceived and discussed. This necessitates culturally sensitive approaches in both diagnosis and treatment [8-10].

Despite the recognized impact of AUB on adolescent health, there remain significant gaps in the research, particularly concerning its prevalence and management in diverse populations. Addressing these research gaps is crucial for improving health outcomes in this vulnerable group. Early intervention is key to preventing the progression of AUB to more severe conditions and enhancing the overall quality of life for affected individuals. Furthermore, educational efforts aimed at increasing awareness of AUB among both patients and healthcare providers are essential for facilitating early detection and improving management outcomes.

Through this study, aside from anovulation, the study aims to evaluate the various causes of menstrual disorders in teenage girls so that they can receive appropriate treatment. Previous studies have primarily focused on heavy menstrual bleeding (HMB), but the current study encompasses all types of menstrual disorders, including infrequent and frequent cycles, heavy and scanty flow, and regular and irregular cycles.

## Materials and methods

This study employed a cross-sectional, observational research design aimed at identifying and analyzing the various etiological factors associated with AUB in adolescent girls. The design was chosen to provide a snapshot of the prevalence and causes of AUB within a specific population, allowing for the collection of a wide range of data that could be used to identify patterns and correlations. The study was conducted over a period of one year at a tertiary care hospital, which ensured access to a diverse and substantial patient population.

The study was conducted in the Department of Obstetrics and Gynecology at Aarupadai Veedu Medical College and Hospital, Pondicherry, India, between November 2022 and May 2024. Ethical approval from Aarupadai Veedu Medical College and Hospital Institutional Ethics Committee (IHEC NO: AV/IHEC/2022/108) was obtained. The teenage girls aged 13 to 18 years who presented with menstrual disorders and who were post-menarche by two years were included in the study. The teenage girls who attended the gynecology opd were evaluated with a detailed and structured history, and appropriate investigations and management were done. The primary objective is to identify the etiological factors of menstrual disorders in teenage girls.

Inclusion criteria for the study were teenage girls post-menarche by two years with menstrual disorders. Exclusion criteria included married girls and those with mental disabilities. The sample size was calculated based on a 17% prevalence of irregular uterine bleeding from a previous study, resulting in a required sample size of 150 participants. Convenience sampling was used for participant selection.

After obtaining a menstrual history and the presenting complaints, a structured history was taken to rule out bleeding diathesis, thyroid dysfunction, eating disorders, and other health issues. Data were collected through a combination of structured interviews, physical examinations, and laboratory investigations. The structured interviews were designed to gather detailed information on the participants' menstrual history, including age at menarche, cycle regularity, duration, and volume of bleeding. Additionally, socio-demographic data, such as age, socioeconomic status, and family history of menstrual disorders, were collected to identify any potential correlating factors. The physical examinations focused on assessing the overall health of the participants, including body mass index (BMI), signs of hirsutism, acne, acanthosis nigricans, and other physical indicators of hormonal imbalances.

Laboratory investigations were conducted to rule out secondary causes of AUB and to identify underlying conditions. These tests included a complete blood count (CBC) to assess for anemia, thyroid function tests to evaluate thyroid disorders, and coagulation profiles to identify any bleeding disorders. In cases where polycystic ovary syndrome (PCOS) was suspected, serum hormone levels, including LH, FSH, and testosterone, were measured. Pelvic ultrasonography was performed in select cases to assess for structural abnormalities such as polyps, fibroids, or ovarian cysts. Abdominal examinations were performed, and hematological investigations were conducted for hemoglobin, hematocrit, platelet count, TSH, bleeding time, and clotting time. If thrombocytopenia was present, prothrombin time and activated partial thromboplastin time were also measured.

Statistical analysis was carried out using IBM SPSS Statistics for Windows, Version 23 (Released 2015; IBM Corp., Armonk, New York, United States). The data were summarized as mean, standard deviation, frequency, and percentage. Descriptive statistics were employed to summarize the demographic characteristics of the study population and the prevalence of different etiological factors. Continuous variables were presented as means with standard deviations, while categorical variables were presented as frequencies and percentages.

## Results

The present study included a total of 150 patients who met the inclusion criteria. The study population had a mean age of 16.64 years, with a standard deviation of ±1.69 years, indicating that most participants were in their mid to late teenage years. The average height among participants was 153.15 cm, with a standard deviation of ±7.84 cm, reflecting a moderate variation in height. The mean weight was 50.52 kg, with a broader standard deviation of ±10.19 kg, showing a significant diversity in body weight within the group. Regarding the age of menarche, participants reported an average onset of 12.00 years, with a standard deviation of ±1.3 years, suggesting that the majority of girls experienced menarche around this age. These physical and developmental characteristics provide an important context for understanding the participants' health profiles in relation to AUB (Table 1).

**Table 1:** Mean level of age, physical character, and age of menarche among participants (N=150)

	Minimum	Maximum	Mean	SD
Age	13.00	18.00	16.64	1.69
Height	130.00	172.00	153.15	7.84
Weight	25.00	78.00	50.52	10.19
Age of menarche	10	15	12.00	1.3

Table 2 presents a detailed analysis of various menstrual and clinical variables among the study participants, offering insights into the distribution and prevalence of different menstrual disorders and related clinical features.

**Table 2:** Distribution of etiological factors associated with abnormal uterine bleeding among the study participants HMB: heavy menstrual bleeding; PCOS: polycystic ovarian syndrome

Etiological characteristics		Frequency	Percent
Menstrual abnormality	Amenorrhea	23	15.3
	HMB	94	62.6
	Scanty bleeding	33	22
Dysmenorrhea	Regular cycles with dysmenorrhea	23	15.3
	Irregular cycles without dysmenorrhea	127	84.7
Regularity	Irregular with amenorrhea	127	85
	regular	23	15
Duration	<8 days	94	63.3
	>8 days	56	36.7
Frequency	<24 days	28	18.6
	24 to 38 days	29	19.3
	>38 days	70	46.6
	Amenorrhea	23	15
Flow	Heavy	96	64
	Normal	21	14
	Scanty	33	22
Thyroid	Hypothyroid-weight gain, cold intolerance, hair loss, constipation	9	6
Bleeding disorder	Bleeding gums and easy bruising	1	0.7
H/o tuberculosis	Cough, evening raise in temperature, loss of appetite	0	0
Clinical hyperandrogenism Insulin resistance	Hirsutism	13	8.6
	Oily face, acne not been controlled	5	3.3
	Acanthosis nigricans	7	4.6
Hemoglobin	Normal	32	21.3
	Mild	42	28
	Moderate	74	49.3
	Severe	2	1.3
Diagnosis	Anovulatory dysfunction	92	61.3
	Hypothyroid	9	6
	Normal	2	1.3
	Ovulatory dysfunction	21	14
	PCOS	25	16.6
	Thrombocytopenia	1	0.6

First, in terms of menstrual abnormalities, the data shows that a significant proportion of participants experienced HMB, which accounted for 94 cases (62.6%). Scanty bleeding was reported in 33 participants (22%), while amenorrhea was observed in 23 participants (15.3%). This indicates that a majority of the participants were dealing with issues related to excessive menstrual bleeding, which could have significant implications for their overall health and well-being. Regarding dysmenorrhea, 23 participants (15.3%) had regular cycles accompanied by dysmenorrhea, while a substantial 127 participants (84.7%) experienced irregular cycles without dysmenorrhea. This suggests that irregular menstrual cycles were far more common in the study group and that these cycles were often not associated with the pain typically observed in regular cycles. The number of pads changed was most common with six pads in 38 participants (25.3%) and two pads in 33 participants (22%).

The regularity of menstrual cycles further emphasizes this pattern, with 128 participants (85%) having irregular cycles associated with amenorrhea, and only 22 participants (15%) maintaining regular cycles. This irregularity is further reflected in the duration and frequency of the menstrual cycles. About 95 participants (63.3%) had menstrual periods lasting less than eight days, while 55 participants (36.7%) experienced longer periods. Furthermore, 70 participants (46.6%) had infrequent menstrual cycles with intervals exceeding 38 days, whereas 28 participants (18.6%) had frequent cycles with intervals of less than 24 days. In terms of menstrual flow, 96 participants (64%) reported heavy flow, 33 participants (22%) had scanty flow, and only 21 participants (14%) experienced what could be considered a normal flow. The clinical evaluation also highlighted issues like hypothyroidism, observed in nine participants (6%), and signs of clinical hyperandrogenism, such as hirsutism (13 participants, 8.6%), oily face with acne (five participants, 3.3%), and acanthosis nigricans (seven participants, 4.6%).

Last, the hematological data revealed that nearly half of the participants (74 participants, 49.3%) had moderate anemia, with a smaller percentage experiencing severe anemia (two participants, 1.3%) or mild anemia (42 participants, 28%). These findings underscore the significant health challenges faced by the teenage participants in the study, particularly in relation to anemia, which is often a consequence of HMB.

## Discussion

Menstrual disorders are common problems among teenage girls and can significantly impact their physical health, emotional well-being, and quality of life. Understanding the etiological factors associated with these disorders is essential for developing effective treatment strategies and providing appropriate care. “Menstrual disorders in teenagers can manifest in various forms, including amenorrhea, heavy and scanty menstrual bleeding, and frequent, infrequent menstrual cycles." Socio-economic factors play a significant role in influencing menstrual health.

The aim of the study was to evaluate etiological factors in association with menstrual disorders among teenage girls, providing a comprehensive analysis of their prevalence, characteristics, and underlying causes. By examining a sample of 150 teenage girls, this study seeks to identify the etiological factors that can help in clinical diagnosis and appropriate management. The findings of this study are expected to enhance our understanding of menstrual health in teenagers and contribute to the development of targeted interventions to improve their overall health and well-being. The majority of teenage girls were less than 16.64±1.69 SD years. The average age of menarche was 12.00±1.3 SD years.

Wasnik et al. study documented menarche occurring at an average age of 13.5 years. About 17% of teenage girls had mid-cycle pain. Adolescent girls had dysmenorrhea in about 81%, with 11% experiencing discomfort, 11% experiencing pain in the lower back, 6.6% experiencing a mild headache, and 2.5% experiencing depression. 3% of adolescent females had no complaints during menstruation [11]. In a different study, the majority of patients needed more pads than the typical amount used each cycle. Fifteen percent needed two precautions. The majority of research patients (63%) had irregular periods, while 17% had no cycle pattern at all. Only 20% of research patients had regular cycles [12]. Omidvar et al. studied that about 73.1% of cycles had a duration of 21-35 days. Twelve percent of them had menstrual flow for longer than seven days, and over half of them had flow for five to six days. Early adolescence was associated with a higher frequency of long blood flow duration than late adolescence. About 30.1% reported excessive blood loss. There was no difference between early and late teenage dysmenorrhea at 66.8%. Menstrual periods are often shorter in early adolescence [13].

In the study by Sharma et al., the subject's average cycle duration was 34.8±11.8 days. There was a significant link observed between the regularity of menstruation and ethnicity, with 167 (64.2%) female participants exhibiting irregular menstrual cycles. Seven females (2.7%), a cycle lasting more than 35 days by 60 females (23.1%) and a typical cycle lasting between 21 and 35 days by 193 females (74.2%) experienced a menstrual period lasting less than 21 days. Of the 231 women, 88.8% had regular menstrual periods. More than half of the girls said they had dysmenorrhea, and it was found that there was a strong correlation between the severity of the condition and the need for therapy and missed school days. Dysmenorrhea was shown to be the most frequent menstruation condition among teenage girls. Menstrual discomfort impacted girls' school absenteeism. Often teenage girls experience severe dysmenorrhea that results in medical intervention [14].

Srivastava et al. documented that the majority of teenage girls (61.25%) reported menstruation issues. Abdominal discomfort was reported as a serious concern by 45.6% of cases of urban girls throughout their menstrual cycle. During the menstrual cycle, 16% and 20.3% of urban and rural females had headaches, respectively. Tiredness has been noticed in 22.4% of rural girls throughout their menstrual cycle. Food cravings were reported by 20.6% and 21.6% of urban and rural females, respectively. When compared to urban girls, a high number of rural girls exhibited psychological-related symptoms [15].

In terms of research results, Hb% was typical in all instances; 33% had 8-10 g%, 65% had 7-8 g%, and 12% had 7 g%. In 6% of individuals, hypothyroidism was found, and all pelvic ultrasounds were normal, concluding that ovulatory dysfunction (AUB-O) was the most frequent cause of unplanned AUB during the post-menarche era [12]. Bieniasz et al. conducted a study for hyperandrogenism; androgen excess was observed in 48 (63.2%) of the patients [16]. In a study by Kushwah et al., the age at menarche was 13.4±0.92. In 93.1% of teenage females, the most prevalent symptom was stomach pain/cramps throughout the menstrual cycle. Dysmenorrhea was the most frequent abnormal condition in teenage girls. Sanitary pads are widely available, and their usage was seen in 83.8% of adolescent girls [17].

From the findings of this study, it is evident that menstrual disorders among teenage girls are multifaceted, with a high prevalence of anovulatory dysfunction (61.3%) after ruling out hypothyroidism (6%), early onset of PCOS (16.6%), coagulation (0.6%), hematological disorders (49.3%), overweight (20%), and obesity (20%). Recommendations include the need for comprehensive screening and diagnostic evaluation to identify and address these disorders early. Healthcare providers should consider a holistic approach, taking into account the diverse etiological factors, to develop treatment plans that will improve the quality of life of teenage girls. Additionally, increasing awareness and education about menstrual health among teenagers and their families can improve early detection and management of these disorders.

Limitations of the study

The cross-sectional nature of the study provides a snapshot in time but does not allow for the assessment of changes or developments in menstrual disorders over time. Longitudinal studies could offer deeper insights into the progression and management of these conditions. The study focused exclusively on adolescent girls aged 13 to 18, which limits the generalizability of the findings to other age groups or populations. Additionally, the study excluded married girls and those with mental disabilities, potentially missing out on variations in menstrual disorders that could occur in these subgroups.

## Conclusions

In conclusion, the present study aimed to evaluate the etiological factors associated with menstrual disorders among teenage girls, encompassing a sample of 150 patients. On clinical evaluation, anovulatory dysfunction was noted; patients had early-onset PCOS (hyperandrogenic anovulation), hypothyroidism, ovulatory dysfunction, and coagulation disorder. In physical parameters, participants were overweight and obese and advised lifestyle modifications. A case of primary thrombocytopenic purpura was observed and treated. Teenage girls with PCOS sensitization have been done for lifestyle modifications and advised to have an optimal BMI. This study helps in the early identification of the etiology of adolescent AUB and their appropriate management of menstrual disorders to improve well-being and enhance reproductive function in the future.
